# Subcellular Localization of a Plant Catalase-Phenol Oxidase, AcCATPO, from *Amaranthus* and Identification of a Non-canonical Peroxisome Targeting Signal

**DOI:** 10.3389/fpls.2017.01345

**Published:** 2017-08-02

**Authors:** Ning Chen, Xiao-Lu Teng, Xing-Guo Xiao

**Affiliations:** State Key Laboratory of Plant Physiology and Biochemistry, College of Biological Sciences, China Agricultural University Beijing, China

**Keywords:** betalain biosynthesis, C-terminal GFP tag, N-terminal GFP tag, non-canonical peroxisomal targeting signal 1 (non-canonical PTS1), plant catalase-phenol oxidase, peroxisome, red amaranth CATPO (AcCATPO), subcellular localization

## Abstract

AcCATPO is a plant catalase-phenol oxidase recently identified from red amaranth. Its physiological function remains unexplored. As the starting step of functional analysis, here we report its subcellular localization and a non-canonical targeting signal. Commonly used bioinformatics programs predicted a peroxisomal localization for AcCATPO, but failed in identification of canonical peroxisomal targeting signals (PTS). The C-terminal GFP tagging led the fusion protein AcCATPO-GFP to the cytosol and the nucleus, but N-terminal tagging directed the GFP-AcCATPO to peroxisomes and nuclei, in transgenic tobacco. Deleting the tripeptide (PTM) at the extreme C-terminus almost ruled out the peroxisomal localization of GFP-AcCATPOΔ3, and removing the C-terminal decapeptide completely excluded peroxisomes as the residence of GFP-AcCATPOΔ10. Furthermore, this decapeptide as a targeting signal could import GFP-10aa to the peroxisome exclusively. Taken together, these results demonstrate that AcCATPO is localized to the peroxisome and the nucleus, and its peroxisomal localization is attributed to a non-canonical PTS1, the C-terminal decapeptide which contains an internal SRL motif and a conserved tripeptide P-S/T-I/M at the extreme of C-terminus. This work may further the study as to the physiological function of AcCATPO, especially clarify its involvement in betalain biosynthesis, and provide a clue to elucidate more non-canonic PTS.

## Introduction

Catalases (EC 1.11.1.6) are highly conserved metalloenzymes that are present in all aerobic and many anaerobic organisms, including bacteria, fungi, plants, and animal cells (Mueller et al., 1997; Engel et al., 2006; Kocabas et al., 2008; Mhamdi et al., 2010; Zhang et al., 2016). They are generally classified into four groups, based on the variety of subunit sizes, the difference of heme prosthetic groups, and the diversity of sequence groups (Zámocký and Koller, 1999; Kocabas et al., 2009), namely (1) monofunctional heme-containing catalases (typical), (2) bifunctional heme-containing catalase-peroxidases, (3) manganese catalases (non-heme-containing catalases), and (4) minor catalases with slight catalytic activities. Their most important function is the decomposition of hydrogen peroxide (H_2_O_2_) into dioxygen and water (Goldberg and Hochman, 1989; Bhaskar and Poulos, 2005; Zámocký et al., 2008; Yuzugullu et al., 2013). The second function of catalases is the oxidation of hydrogen donors such as ethanol, formic acid, phenols and methanol, with the concomitant consumption of peroxide, which is the characteristics of catalase-peroxidases (Levy et al., 1992; Fraaije et al., 1996; Kocabas et al., 2009; Loncar and Fraaije, 2015). A new function of catalases, the oxidase activity in the absence of hydrogen peroxide, was discovered by Vetrano et al. (2005) on human catalases and bovine liver catalases, although the enzyme was not further characterized. Later on, Kocabas et al. (2008) characterized this type of enzyme from the thermophilic fungus *Scytalidium thermophilum*, and demonstrated its peroxide-independent phenol oxidase activity, resembling that of catechol oxidases, but also possessing some features of laccases. Thus, Kocabas et al. (2008) named it catalase-phenol oxidase (CATPO). This phenol oxidase activity was confirmed and the nature of the phenol oxidation, similar mainly to those of laccases, was revealed by Avci et al. (2013) via analysis of its spectrum of substrates that were oxidized. Besides *S. thermophilum* catalase, some other heme-containing catalases, such as those from *Aspergillus niger*, human erythrocytes, and bovine liver, also display more or less phenol oxidase activity (Kocabas et al., 2008). Recently, Baginski and Sommerhalter (2017) reported that a manganese catalase from thermophilic bacterium, *Thermomicrobium roseum*, showed phenol oxidase activity. We previously identified a CATPO from a betalain-producing plant, red amaranth (*Amaranthus cruentus*) (Teng et al., 2016). Unlike microbial and mammalian CATPOs, this plant CATPO (AcCATPO) exhibited both monophenolase activity toward L-tyrosine and diphenolase activity toward L-3,4-dihydroxyphenylalanine (L-DOPA), in addition to the classical catalase activity toward H_2_O_2_, i.e., catalase-tyrosinase activity.

The tyrosinase (EC 1.14.18.1; EC 1.10.3.1) has long been proposed to be involved in the hydroxylation of tyrosine to form L-DOPA, the first step in the biosynthesis of betalains (Mueller et al., 1996; Steiner et al., 1996; Strack et al., 2003; Han et al., 2009; Harris et al., 2012; Gandia-Herrero and García-Carmona, 2013). Although this step, recently proven at the molecular level to be catalyzed by sugar beet CYP76AD1, CYP76AD5, and CYP76AD6 (DeLoache et al., 2015; Polturak et al., 2016; Sunnadeniya et al., 2016), the involvement of tyrosinase seemly can not be excluded, because the co-expression of a tyrosinase gene from non-betalain-producing mushroom (*Lentinus edodes*) with a DODA gene from betalain plant (*Mirabilis jalapa*) resulted in formation of betaxanthins in the cell cultures of non-betalain plants, tobacco and Arabidopsis (Nakatsuka et al., 2012). Thus the question raised is whether the AcCATPO, which possesses both tyrosinase activity and catalase activity, may also be involved in the hydroxylation of tyrosine, the first step of betalains biosynthesis? Are there other, even general functions of this enzyme? To address such questions, we started to investigate the subcellular localization of AcCATPO, because the subcellular compartment of a protein is often linked to its function (Grefen et al., 2008; Bononi and Pinton, 2015; Itzhak et al., 2016; Xiong et al., 2016). Here, we report on the subcellular localization of AcCATPO by combing a bioinformatics approach with GFP tagging at the N-terminal and C-terminal of AcCATPO and identification of a non-canonical targeting signal by deleting the C-terminal tripeptide and decapeptide of the AcCATPO and using the decapeptide to guide GFP to the expected organelle.

## Materials and Methods

### Gene and Plant Materials

*AcCATPO* (GenBank accession KP710221) was previously cloned in our group (Teng et al., 2016). The mCherry-peroxisome-localized marker, pBin20-mCherry-PTS1 (Li et al., 2014), was generously donated by Dr. W.-C. Yang (Institute of Genetics and Developmental Biology, Chinese Academy of Sciences, Beijing, China). Binary vectors carrying 35S::N-GFP (Zhao et al., 2011) or 35S::C-GFP (Zhao et al., 2015) were kindly provided by Drs. C.-Q. Sun and Y. Guo (China Agricultural University, Beijing, China), respectively. The mitochondrion-selective fluorescent stain, MitoTracker Deep Red FM, was purchased from Thermo Fisher Scientific (Waltham, MA, United States).

Plants of tobacco (*Nicotiana benthamiana*) were grown at 24 ± 2°C under 16 h light/8 h dark cycle with a light intensity of ca. 100 μmol m^-2^ s^-1^ in a culture room.

### Construction of Vectors

A DNA fragment consisting of the coding sequence of the C-terminal 10 amino acid residues (decapeptide: ASRLNVRPTM) of AcCATPO, with respective *Eco*R I and *Kpn* I restriction sites at the 5′- and 3′-end, was synthesized by Sangon (Shanghai, China) and named 10aa.

The coding sequences of the C-terminal tripeptide (PTM) and decapeptide of AcCATPO were PCR-deleted with specific primer pairs (**Table 1**), and the deleted versions were designed as *AcCATPO*Δ*3* and *AcCATPO*Δ*10*, respectively.

**Table 1:** PCR primers and synthesized 10aa gene fragment used in this study.

	Sequence (5′–3′)	Restriction sites
**Primer name**		
CATPOGFP-F	CGAGCTCATGGATCCTTACAAGTA	*Sac* I
CATPOGFP-R	CGTCGACCATGGTTGGTCTTATGTTAAGTC	*Sal* I
CATPOΔ3GFP-R	CGTCGACTCTTATGTTAAGTCTA	*Sal I*
CATPOΔ10GFP-R	CGTCGACCACTTTCATACCCAGAG	*Sal I*
GFPCATPO-F	GGAATTCATGGATCCTTACAAGTAT	*Eco*R I
GFPCATPO-R	GGGTACCTCACATGGTTGGTCTTATGTTAAGTC	*Kpn* I
GFPCATPOΔ3-R	GGGTACCTCATCTTATGTTAAGTCTA	*Kpn* I
GFPCATPOΔ10-R	GGGTACCTCACACTTTCATACCCAGAG	*Kpn I*
**DNA name**		
10aa	GAATTCGCAAGTAGACTTAACATAAGACCAACCATGTGAGGTACCA	*Eco*R I/*Kpn* I
	ACTTAAGCGTTCATCTGAATTGTATTCTGGTTGGTACACTCCATGG

The full-length coding sequence and the deleted versions of *AcCATPO* with or without stop codon were PCR-amplified by using gene-specific primer pairs (**Table 1**), linked to a pGEM-T vector (Promega, United States) and sequenced for authenticity. Then, the coding sequences without stop codon were inserted into p35S::C-GFP and those with stop codon, into p35S::N-GFP vectors in frame with correspondent restriction enzymes (**Table 1**) to generate vectors p35S::AcCATPO-GFP, p35S::AcCATPOΔ3-GFP, p35S::AcCATPOΔ10-GFP and p35S::GFP-AcCATPO, p35S::GFP-AcCATPOΔ3 and p35S::GFP-AcCATPOΔ10, respectively. For constructing p35S::GFP-10aa, the synthesized DNA fragment (10aa sequence) was first ligated to the pGEM-T vector, and sequenced. Then the 10aa sequence was taken out with *Eco*R I and *Kpn* I, and inserted into p35S::N-GFP cut by *Eco*R I and *Kpn* I.

### Plant Transformation and Fluorescence Detection

The peroxisomal marker, pBin20-mCherry-PTS1, and all GFP constructs were transformed into *Agrobacterium tumefaciens* strain GV3101. For transient expression of single construct, the resultant bacterial suspension was directly infiltrated into young leaves of tobacco (*N. benthamiana*) as previously reported (Han et al., 2013). For co-expression of the fused GFP and peroxisomal marker RFP, the bacterial suspension harboring GFP construct and that carrying peroxisomal RFP marker were mixed in a ratio of 1:1, and then the resulting mixture was co-infiltrated into young leaves as reported before (Chen et al., 2017). Two to 3 days after infiltration, the abaxial epidermis of the leaves was observed for fluorescence under Carl Zeiss 710 confocal laser scanning microscopy (CLSM). The GFP and RFP channels were acquired and the fluorescence signals were detected as previously described (Chen et al., 2017). MitoTracker Deep Red staining of GFP-construct transgenic tobacco leaves was performed according to the manufacturer’s instructions, and the far red-fluorescent signal was detected using an emission bandwidth of around 665 nm after scanning at an excitation wavelength of 644 nm. The images were processed with Zeiss LSM Image Browser software and then the combined pictures were generated with Photoshop.

### Database Searches and Bioinformatics Prediction

The whole amino acid sequence of AcCATPO was used for database searches and bioinformatics prediction. The following on-line prediction programs were consulted: targeting signal prediction programs TargetP1.1^1^, ChloroP1.1^2^, SignalP4.1^3^, Protein Prowler^4^, PredSL^5^, and PredPlantPTS1 and subcellular localization prediction programs WoLF PSORT II^6^, ProtComp 9.0^7^, UniProtKB^8^, and CELLO V2.5^9^. For each program, the default “cut-off” and organism “Plant,” or “Eukaryotes,” was chosen if “Plant” was not available.

## Results

### Bioinformatics Prediction of Targeting Signal and Subcellular Localization

Before performing experimental assays, we consulted six on-line bioinformatics “individual predictors” and four “integrators” programs widely used to predict targeting signal and subcellular localization of AcCATPO, respectively, by using the whole amino acid sequence. No targeting signal of any types was predicted in AcCATPO with all six individual predictors used (Supplementary Table S1). However, a peroxisomal localization of AcCATPO was forecasted by all integrators programs except UniProt, which did not contain any information on this protein (**Table 2** and Supplementary Table S1). These prediction results implied that the AcCATPO might have some ways different from known canonical peroxisomal targeting signal (PTS, including PTS1 and PTS2) to target to peroxisomes.

**Table 2:** Comparison of targeting signals and subcellular localizations of AcCATPO predicted and N- and C-terminal GFP tagged.

Gene name	Length (AA)	Cell localization (this study)		Cell localization- bioinformatic prediction									
		C-TERM	N-TERM	WoLF PSORT II_Over 2			ProtComp 9.0 _ Over 1					CELLO V2.5	
							LDB	PLDB	NN	PT	ALL	LOC	RC
AcCATPO	492	NUC/CYT/PER	NUC/PER	PER	7	PER	3.1	1.2	0.96	8.12	3.53	PER	4.0
				MIT	4	EC	1.9	0.7	0.96	0.05	2.28	MIT	0.4
				CHL	2	CYT	2.0	1.2	0	0	2.02	CYT	0.2
						MIT	3.0	0	0	0	2.00		

*AA, amino acids; C-TERM, C-terminal-tagged GFP; N-TERM, N-terminal-tagged GFP; NUC, nucleus; CYT, cytoplasm; PER, peroxisome; MIT, mitochondria; CHL, chloroplast; EC, extra cellular; LDB, localization from database; PLDB, predicted location from database; NN, neural networks; PT, pentamers; ALL, combined results from LDB, PLDB, NN, and PT; LOC, prediction of location; RC, reliability class*.

**FIGURE 1:**
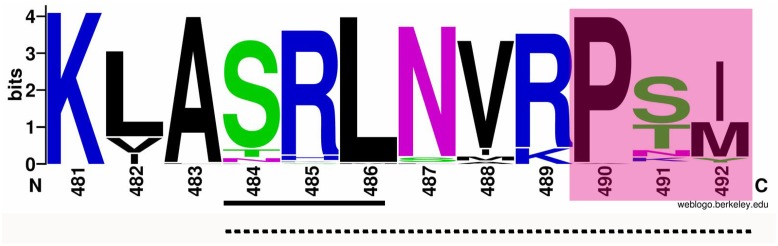
Web-logo image of the C-termini of AcCATPO and plant catalases selected. The amino acid sequences of plant catalases selected were the same as previously reported (Teng et al., 2016). The C-terminal 12 amino acid sequences of AcCATPO and common plant catalases selected were subjected to weblogo website (http://weblogo.berkeley.edu/logo.cgi). Underlined with solid line, Conserved SRL motif; Underlined with dash line; Putative non-canonical peroxisomal targeting signal 1; Shaded, Conserved C-terminal extreme tripeptide.

To analyze possible non-canonical PTS and/or some other motif(s) to import AcCATPO to peroxisomes, we compared the AcCATPO amino acid sequence with those of common plant catalases retrieved from the NCBI database, as reported before (Teng et al., 2016), with focus on the carboxyl terminus (C-terminus). We were able to identify a conserved internal SRL motif, which is located in a distance of nine amino acids from the C-termini of almost all catalases compared (Supplementary Figure S1 and **Figure 1**, underlined). The web-logo analysis of the last 12 amino acid residues from the C-terminus showed a remarkable conservation of the residues around the SRL motif, especially with the C-terminal tripeptide P-S/T-I/M (i.e., poline-polar-non-polar) (**Figure 1**, shaded). This analysis suggested the AcCATPO might use a peptide fragment of these 10–12 amino acids, as a non-canonical PTS1, to target the enzyme to peroxisomes.

### Tagging GFP at the N-terminus of AcCATPO and Its Mutants

In order to verify bioinformatics-based prediction and PTS analysis results, we carried out subcellular targeting experiments *in vivo*, according to “the gold standard for studying protein localization in peroxisomes to date” (Reumann et al., 2016). First, we deleted the coding regions of C-terminal tripeptide (PTM) and then decapeptide (ASRLNVRPTM) from *AcCATPO* and generated *AcCATPO*Δ*3* and *AcCATPO*Δ*10*, respectively, to weaken or abolish the putative non-canonical PTS1 analyzed above. Then we fused GFP at the N-termini of AcCATPO, AcCATPOΔ3, and AcCATPOΔ10 and transiently expressed the fusion protein under the control of the constitutive CaMV 35S promoter (35S) in the epidermal leaf cells of tobacco (*N. benthamiana*), with the peroxisomal marker, pBin20-mCherry-PTS1, and the empty vector, p35S::N-GFP, as controls. The subcellular localization of GFP and marker RFP was analyzed with a Carl Zeiss 710 CLSM. Prior to proceeding further, however, we verified, the punctum-like red fluorescence signal in the peroxisomes of abaxial epidermal cells of the pBin20-mCherry-PTS1-transgenic tobacco leaves distinguishable from that of the MitoTracker Deep Red, a mitochondrion-selective fluorescent dye (Supplementary Figure S2). With this putative confusion being clarified, we went ahead and used the pBin20-mCherry-PTS1 as the peroxisomal marker RFP in the following GFP tag experiments.

**FIGURE 2:**
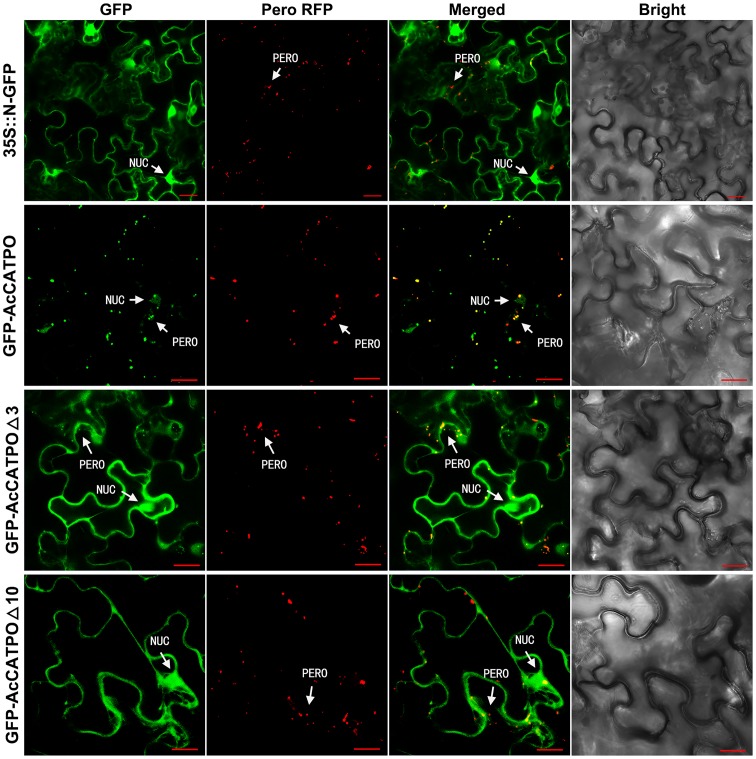
Confocal fluorescence scanning of AcCATPO and AcCATPOΔ with N-terminal GFP tag (GFP-Gene). Each GFP construct and peroxisomal RFP marker were co-transformed to tobacco (*Nicotiana benthamiana*) leaf epidermal cells by agro-infiltration, and 2 or 3 days after infiltration, the cells were subjected to Carl Zeiss 710 confocal laser scanning microscopy for fluorescence detection. NUC, nucleus; PERO, peroxisome; Pero RFP, Peroxisome-localized RFP marker; Δ3 and Δ10, C-terminal 3 or 10 amino acids deleted, respectively; GFP-AcCATPO, GFP-AcCATPOΔ3 and GFP-AcCATPOΔ10, GFP tagged at the N-terminus of the protein; Bar = 20 μm.

In transgenic tobacco epidermal leaf cells, unfused N-GFP was evenly distributed in the nucleus and cytoplasm (**Figure 2**, 35S::N-GFP), while the peroxisomal marker RFP, present as red puncta, resided only in the peroxisomes (**Figure 2**, Pero RFP). For the GFP-AcCATPO fusion protein, it targeted to the nucleus and the peroxisome (**Figure 2**, GFP-AcCATPO), and its GFP signal in the peroxisomes was overlapped with the peroxisomal marker RFP, with a colocalization efficiency of ca. 43.4%, when co-expressed with the marker (**Figure 2**, GFP-AcCATPO), indicating peroxisomal targeting of the fusion protein. When the C-terminal tripeptide was deleted, the fusion protein GFP-AcCATPOΔ3 was addressed not only to the nucleus and the peroxisome, but also to the cytosol (**Figure 2**, GFP-AcCATPOΔ3), indicating that the tripeptide PTM at the extreme C-terminus played an important role in sorting or rapid sorting the enzyme to peroxisomes. It was noted that although GFP-AcCATPOΔ3 appeared to be present in peroxisomes, the number of peroxisomes with a GFP signal (ca. 29.4%) appeared inferior to those obtained with wild GFP-AcCATPO. In addition, its GFP signals in the cytosol were diffusive and weaker than those in the nuclei. When the C-terminal decapeptide was deleted, the fusion protein GFP-AcCATPOΔ10 was confined exclusively to the cytosol and the nucleus, and no more GFP signal was detected in the peroxisomes, which was confirmed by the absence of overlay between the GFP and the peroxisomal marker RFP (**Figure 2**, GFP-AcCATPOΔ10).

These N-GFP tagging results demonstrated that AcCATPO was localized to the nucleus and the peroxisomes, and this peroxisomal localization is partly attributed to the C-terminal tripeptide, and completely attributed to the C-terminal decapeptide.

### Tagging GFP at the C-termini of AcCATPO and Its Mutant

In order to further verify the peroxisomal localization of AcCATPO and validate the contribution of its C-terminal to this localization, we alternatively spliced GFP to the C-terminus of AcCATPO, AcCATPOΔ3 and AcCATPOΔ10 and expressed the fusion proteins under the control of 35S promoter in tobacco leaf epidermal cells, as for N-GFP tagging, with the peroxisomal marker and the p35S::C-GFP (empty vector) serving as controls. As in the N-GFP tagging, the leaf epidermal cells were examined by CLSM with Zeiss 710 for green and red fluorescence.

**FIGURE 3:**
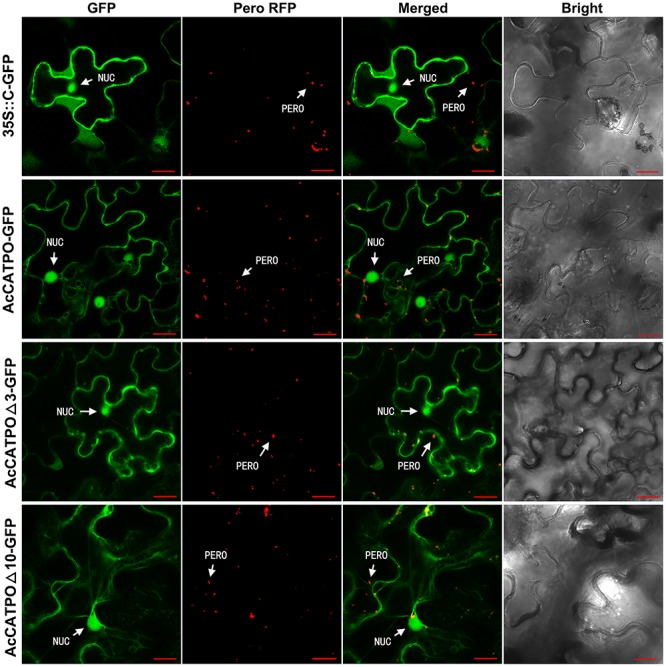
Confocal fluorescence scanning of AcCATPO and AcCATPOΔ with C-terminal GFP tag (Gene-GFP). See **Figure 2** legend for detail, except AcCATPO-GFP, AcCATPOΔ3-GFP and AcCATPOΔ10-GFP, GFP tagged at the C-terminus of the protein.

Identical to free N-GFP, the free C-GFP protein was uniformly localized to the nucleus and cytosol in transgenic leaf epidermal cells (**Figure 3**, 35S::C-GFP). However, different from N-GFP fusion protein, the AcCATPO-GFP fusion protein targeted mainly to the nucleus and the cytosol, and only few peroxisomes showed a green fluorescence signal, which could coincide with the red one from peroxisome-localized RFP (**Figure 3**, AcCATPO-GFP), signifying a heavy interference of the peroxisomal localization. Similarly, the fusion protein AcCATPOΔ3-GFP resided primarily in the nucleus and the cytosol, and it was hard to see any peroxisomes that displayed a GFP signal co-localizing with the peroxisomal marker RFP (**Figure 3**, AcCATPOΔ3-GFP). In line with AcCATPOΔ3-GFP, the GFP signal of the fusion protein AcCATPOΔ10-GFP was recorded almost only in the nucleus and the cytosol, but nearly not in the peroxisomes (**Figure 3**, AcCATPOΔ10-GFP).

The results outlined above, indicated that fusing GFP at the C-terminus of AcCATPO shifted its peroxisomal localization to the cytosol, without changing its nuclear localization, no matter the C-terminal tripeptide and decapeptide were deleted or not, and thus confirming the contribution of the C-terminal of AcCATPO, especially the free and whole C-terminal, to its peroxisomal localization.

### Guiding GFP by the C-terminal Decapeptide of AcCATPO

To further confirm the C-terminal decapeptide of AcCATPO as a non-canonical PTS1 to import enzymes to peroxisomes, we fused the decapeptide at the C-terminus of GFP and expressed the fusion protein GFP-10aa in tobacco as we did for the N-GFP, not only with the peroxisomal marker RFP, and the empty vector, p35S::N-GFP, but also with MitoTracker Deep Red as controls. The GFP and RFP as well as far-red fluorescence signals were detected as described above.

**FIGURE 4:**
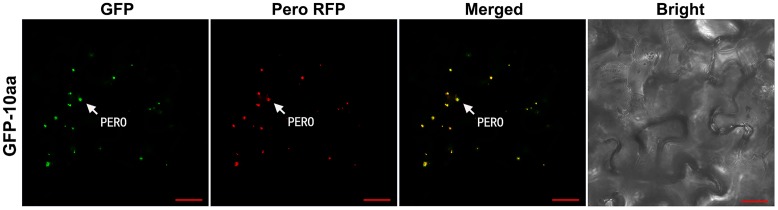
Confocal fluorescence scanning of GFP guided by the C-terminal decapeptide of AcCATPO. See **Figure 2** legend for tobacco leaf agro-infiltrating transformation and fluorescence detection, except the leaf cells were stained, before the detection, with MitoTracker Deep Red FM according to the manufacturer’s instruction. PERO, peroxisome; Pero RFP, Peroxisome-localized RFP marker; Bar = 20 μm.

In the leaf cells of transgenic tobacco plants, the green fluorescent signal from the fusion protein GFP-10aa was exclusively detected in the peroxisomes, but neither in other organelles such as mitochondria, nor in the cytosol, which was demonstrated by the full co-localization of the GFP signal with the RFP signal from the peroxisomal marker (**Figure 4**).

## Discussion

In order to elucidate the physiological functions of AcCATPO, the first catalase-phenol oxidase identified *in planta* (Teng et al., 2016), we started the work by investigating its subcellular localization, since a protein, once biosynthesized in cells, is transported to a specific subcellular compartment to perform its function (Zhang et al., 2007; Grefen et al., 2008; Li et al., 2014; Bononi and Pinton, 2015; Reumann et al., 2016).

Following the recommendation of Tanz and Small (2011), we used the TargetP and other five “individual predictors” to predict possible targeting signals in AcCATPO, including PredPlantPTS1 (Lingner et al., 2011; Reumann et al., 2012), which was recommended for PTS1-specific prediction in plants by Cross et al. (2016). None of these widely used individual predictor programs indicated the putative presence of a targeting signal in AcCATPO (Supplementary Table S1). We then consulted four “integrators” commonly used for subcellular localization prediction. Intriguingly, three of four integrators, WoLF PSORT II, ProtComp 9.0 and CELLO V2.5 targeted the AcCATPO to peroxisomes, albeit the integrator UniProtKB did not yield any useful result (**Table 2** and Supplementary Table S1), probably due to the lack of data on AcCATPO in the corresponding data bank.

It is well known that the sorting of proteins to peroxisomes mainly depends on either of two types of signal sequences, PTS1 and PTS2 (see Rucktäschel et al., 2011; Baker et al., 2016; Erdmann, 2016 for reviews and references within). The PTS1 as the most common one, is a tripeptide present at the C-termini of proteins and frequently ends with the sequence S-K-L or its variants with a consensus sequence S/A/C-K/R/H-L/M (Gould et al., 1989; Lametschwandtner et al., 1998; Brocard and Hartig, 2006; Williams et al., 2012; see Dias et al., 2016; Meinecke et al., 2016 for reviews and references within). In contrast to PTS1, the PTS2 functions at internal locations, with a conserved non-apeptide in the N-terminal domain, such as R-L-X5-H-L, and R/K-L/V/I-X5-H/Q-L/A (X: any amino acid) (Terlecky et al., 1995; Petriv et al., 2004; see Baker et al., 2016; Meinecke et al., 2016 for reviews and references within).

In plants like *Arabidopsis thaliana*, the PTS1 and PTS2 are used by about 70 and 30% of the known peroxisomal proteins, respectively (Reumann, 2004). However, the plant PTS1 and/or PTS2 is more or less distinct from typical PTS1 and/or PTS2 (Kamigaki et al., 2003; Oshima et al., 2008; Reumann, 2011; Chowdhary et al., 2012; Lingner et al., 2012; Ramirez et al., 2014; see Cross et al., 2016; Young and Bartel, 2016 for reviews and references within). According to Reumann et al. (2016), almost all experimentally verified plant PTS1 tripeptides identified to date have the following pattern: one low-abundance PTS1 residue (denoted as *x, y*, or *z*) is combined with two high-abundance PTS1 tripeptide residues (*x*-K/R-L/M/I, S/A-*y*-L/M/I, S/A-K/R-*z*), and at least 35 functional plant PTS1 tripeptide residues have been reported. Plant PTS2 is located near the N-terminus of peroxisomal proteins and is defined by the loose consensus sequence R-L/I/Q-X5-H-L or its extended one, R/K-L/V/I-X5-Q/H-L/A (X: any amino acids) (Glover et al., 1994; Reumann, 2004).

Even with those PTS consensus sequences in calculation, all six individual predictor used, including plant PTS1-specific predictor PredPlantPTS1, failed to forecast any kind of PTS in AcCATPO, whereas the AcCATPO was predicted to localize to the peroxisome by 3 integrators predictors (**Table 2** and Supplementary Table S1). Our comparison of amino acid sequences between AcCATPO and common plant catalases, a group of the abundant enzymes in peroxisomes (Reumann et al., 2016), achieved identification of a SRL motif of canonical PTS1 within nine amino acid residues from the C-terminus and a conserved tripeptide P-S/T-I/M at the extreme of C-terminus in AcCATPO, with a consensus sequence S-R-L-N-I-R-P-T-M (**Figure 1** and Supplementary Figure S2). This kind of C-terminal internal SRL motif and C-terminal extreme P-S/T-I/M tripeptide were reported in some plant catalases (Suzuki et al., 1994; Willekens et al., 1995) and are conserved in nearly all plant catalases analyzed in this study (**Figure 1** and Supplementary Figure S1), suggesting that the AcCATPO, as classical plant catalases, may use the C-terminal internal SRL motif and C-terminal P-S/T-I/M tripeptide as a non-canonical PTS1 signal to sort to peroxisomes.

In order to validate the above-discussed non-canonical PTS1 hypothesis and to somehow arbitrate the quality of software-based predictions, we chose to apply *in vivo* subcellular targeting analyses. This approach is advantageous as compared to other plant subcellular localization methods such as cell fraction and immunohistochemical method (Geldner et al., 2009; Tanz et al., 2013; Reumann et al., 2016). Our experimental results with GFP tagging at the N-terminus of AcCATPO demonstrated that the fusion protein GFP-AcCATPO was indeed localized to the peroxisome as well as to the nucleus (**Figure 2**, GFP-AcCATPO). The peroxisomal localization of the GFP-AcCATPO is in agreement with the predication by three integrators, WoLF PSORT II, ProtComp 9.0 and CELLO V2.5 (**Table 2**), indicating the importance of the C-terminal in peroxisomal localization. This importance was reinforced by the fact that deletion of the tripeptide P-T-M at the extreme of C-terminal end almost abolished peroxisomal localization but not that one in nuclei of AcCATPO (**Figure 2**, GFP-AcCATPOΔ3), and removing the C-terminal decapeptide excluded the peroxisomes but not the nuclei as the residence of AcCATPO (**Figure 2**, GFP-AcCATPOΔ10). Thus, our above hypothesis that the AcCATPO uses the non-canonical PTS1 signal in the C-terminal to sort to peroxisomes is validated, and at the same time, the probability that the AcCATPO might also be imported into tobacco peroxisomes by heterooligomerization with tobacco endogenous CAT and piggy-back import is excluded. Nevertheless, we note the report of Kamigaki et al. (2003) that reads the tripeptide P-S-I, at the extreme C-terminus of pumpkin catalase 1 is unnecessary for targeting, but an internal PTS1-like sequence, Q-K-L, at position -13 to -11 from the C-terminus, is essential for targeting to peroxisomes.

To further verify the vital role of the non-canonical PTS1 signal in the C-terminal to the peroxisomal localization of AcCATPO, we “masked” the C-terminal by fusing the GFP at the C-terminus of AcCATPO. As expected, the fusion protein AcCATPO-GFP nearly did not target to the peroxisome indeed, but to the cytosol instead (**Figure 3**, AcCATPO-GFP). Similar, if not identical results were also observed for the fusion proteins AcCATPOΔ3-GFP and AcCATPOΔ10 (**Figure 3**, AcCATPOΔ3-GFP and AcCATPOΔ10-GFP). These results indicate that just like deleting the non-canonical PTS1, masking the non-canonical PTS1 signal-containing C-terminus can lead to mislocalization of AcCATPO, and thus further confirms that the non-canonical PTS1 signal is responsible for peroxisome targeting of AcCATPO. Huh et al. (2003) observed that proteins localized to the peroxisome and endoplasmic reticulum (ER), which often contain C-terminal targeting signals, were mislocalized due to the C-terminal GFP, while Simpson et al. (2000) demonstrated that most signal peptides located at the N-terminus of human proteins were masked by the N-terminal GFP fusion. This may explain the discrepancy in peroxisomal localization between N- and C-terminal GFP tagging of AcCATPO (**Figure 2** vs. **3**). The disagreement between N-terminal and C-terminal tagging of a protein in subcellular localization was also reported by Thornton et al. (2011) for ZnT5vA, by Palmer and Freeman (2004) for eight proteins with various functions, and by Reuter et al. (2016) for *Trichoderma reesei* hydrophobin HFBII and for *Fusarium verticillioides* HYD3 and HYD4.

Above “lost of function” results and discussion demonstrated clearly that the C-terminal non-canonical PTS1 of AcCATPO is responsible for importing the AcCATPO to the peroxisomes. This role of the C-terminal non-canonical PTS1 was further strengthen by the “gain of function” results: it did import the fusion protein GFP-10aa to the peroxisomes exclusively (**Figure 4**).

It is worth noting that GFP tagging at the N- and C-termini of AcCATPO and its mutants (AcCATPOΔ3 and AcCATPOΔ10) shared same localization in the nucleus (**Figures 2**, **3**). However, this nuclear localization of AcCATPO as well as its mutant forms was not predicted by either individual predictors or integrators used (**Table 2** and Supplementary Table S1). The reason for this kind of divergence between informatics prediction programs and experimental data remains to be exploited, as the discrepancy has been reported by larger number of investigators, such as Nelson et al. (2007), Tanz and Small (2011), and Xiong et al. (2016).

Given that the sugar beet CYP76AD1 and CYP76AD6, which have been verified at the molecular level as the major enzymes responsible for the hydroxylation of tyrosine of betalain biosynthesis (Polturak et al., 2016; Sunnadeniya et al., 2016), is localized in the nucleus and the cytosol (Chen et al., 2017), it is plausible that the nucleus-localized AcCATPO, may be involved in betalain biosynthesis also via hydroxylation of tyrosine, as proposed by Teng et al. (2016).

## Conclusion

AcCATPO is localized to the peroxisome as well as to the nucleus when expressed in tobacco leaf cells and the peroxisomal localization is directed by the C-terminal non-canonical PTS1, (A)-S-R-L-N-I-R-P-T-M. Revelation of the subcellular compartmentation of AcCATPO, may expedite study of its physiology function, and especially clarify its involvement in betalain biosynthesis, and identification of the non-canonic PTS1 from this plant catalase-phenol oxidase may provide a new clue to search more non-canonic and/or atypical peroxisome targeting signals.

## Author Contributions

X-GX, NC, and X-LT planned and designed the studies. NC performed experiments. NC and X-GX analyzed the data. NC, X-LT, and X-GX wrote the manuscript.

## Conflict of Interest Statement

The authors declare that the research was conducted in the absence of any commercial or financial relationships that could be construed as a potential conflict of interest.

## Footnotes

1 http://www.cbs.dtu.dk/services/TargetP

2 http://www.cbs.dtu.dk/services/ChloroP

3 http://www.cbs.dtu.dk/services/SignalP/

4 http://bioinf.scmb.uq.edu.au:8080/pprowler_webapp_1-2/

5 http://aias.biol.uoa.gr/PredSL/

6 http://www.genscript.com/wolf-psort.html

7 http://www.softberry.com

8 http://www.uniprot.org

9 http://cello.life.nctu.edu.tw/

## Abbreviations

Gene-GFP: GFP tagged at the C-terminus of the gene
GFP: green fluorescent protein
GFP-Gene: GFP tagged at the N-terminus of the gene
RFP: red fluorescent protein.
