# Supplementary material for: Subcellular Localization of a Plant Catalase-Phenol Oxidase, AcCATPO, from Amaranthus and Identification of a Non-canonical Peroxisome Targeting Signal
Source: Front Plant Sci. 2017 Aug 2;8:1345. doi: 10.3389/fpls.2017.01345 (PMC5539789; doi:10.3389/fpls.2017.01345)
Supplement: Supplementary file 1 [file Data_Sheet_1.PDF]

Supplementary Material

Subcellular localization of a plant catalase-phenol oxidase, AcCATPO, from *Amaranthus* and identification of a non-canonical peroxisome targeting signal

Ning Chen, Xiao-Lu Teng, Xing-Guo Xiao*

***: Correspondence**

Xing-Guo Xiao, xiaoxg@cau.edu.cn; xiaoxg06@gmail.com

1 Supplementary Tables

Table S1 Bioinformatic prediction of signal peptide and subcellular localization of AcCATPO

	Signal Peptide												Cell Localization- Bioinformatic Prediction										
Gene name	TargetP		SignalP			CholoP		PredSL	PredPlantPTS1		Protein Prowler		WoLF PSORT II _ Over 2		ProtComp 9.0 _ Over 1						Uni Prot	CELLO V2.5	
	LOC	RC	POS	VAL	SIG	cTP	SCR	LOC	SCR	PTS1 Domain	LOC	PTS1 Prowler				LDB	PLDB	NN	PT	ALL	LOC	LOC	RC
AcCATPO	O	3	1-157	0.126	N	N	0.457	O	0.036	N	O	0.00	PER	7	PER	3.1	1.2	0.96	8.12	3.53	/	PER	4.0
													MIT	4	EC	1.9	0.7	0.96	0.05	2.28		MIT	0.4
													CHL	2	CYT	2.0	1.2	0	0	2.02		CYT	0.2
															MIT	3.0	0	0	0	2.00			

AA, amino acids; LOC, prediction of location; O, any other location (expect cTP, mTP and SP); RC, reliability class; POS, position; VAL, value; SIG, signal peptide; N, no; cTP, chloroplast transit peptide; SCR, score; PTS1 Domain, peroxisome targeting signal type 1 domain; PTS1 Prowler, PTS1 predictor; PER, peroxisome; MIT, mitochondria; CHL, chloroplast; EC, extra cellular; CYT, cytoplasm; LDB, localization from database; PLDB, predicted location from database; NN, neural networks; PT, pentamers; ALL, combined results from LDB, PLDB, NN and PT.

2 Supplementary Figures

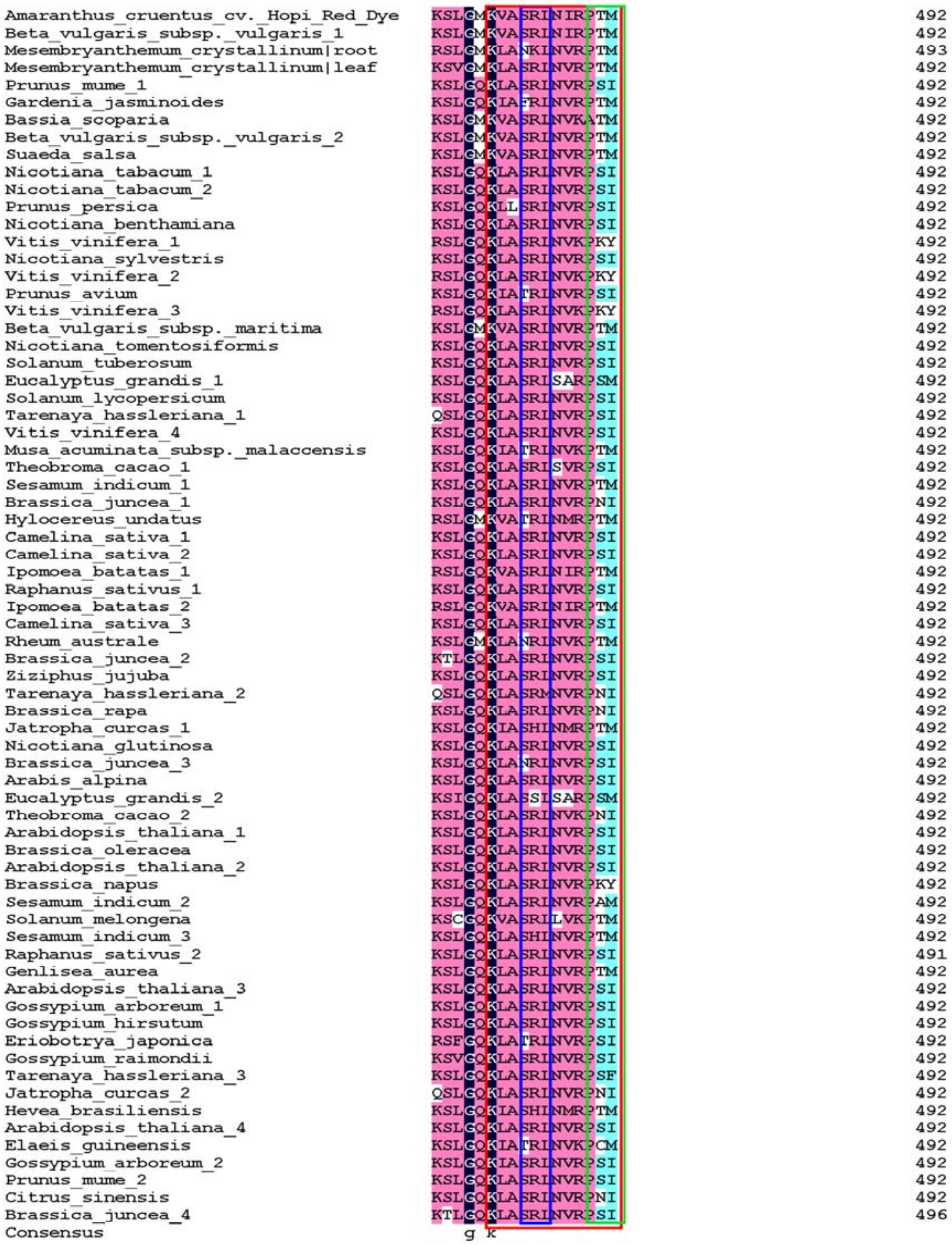

Fig. S1. Comparison of the C-terminals of AcCATPO with plant catalases

Red boxed, Weblogo analysis region; Blue boxed, SRL motif; Green boxed, Tripetide at the extreme

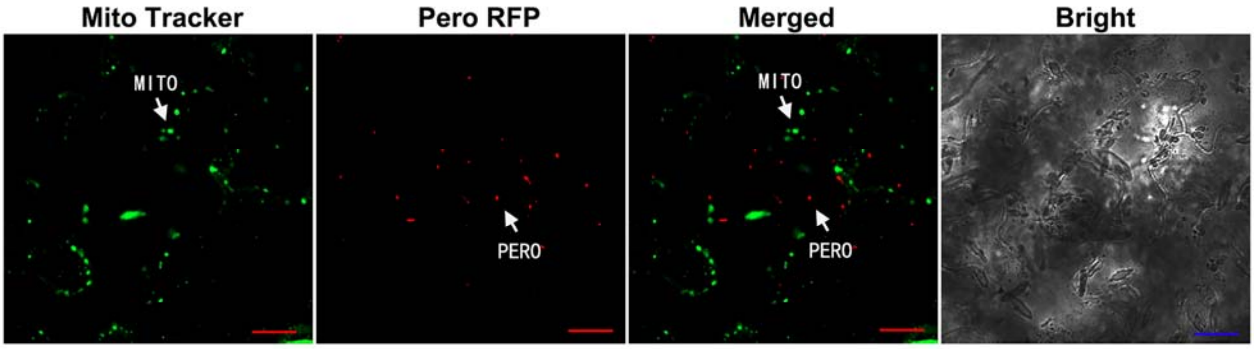

Figure S2 Comparison of confocal fluorescence image of peroxisomal marker RFP with that of MitoTracker Deep Red-stained mitochondria in the leaf cells of transgenic tobacco (*Nicotiana benthamiana*)

MITO, mitochondrion; MitoTracker, MitoTracker Deep Red FM; PERO, peroxisome; Pero RFP, Peroxisome-localized RFP marker; Bar=20 μm.
